# Pre-Existing Isoniazid Resistance, but Not the Genotype of *Mycobacterium Tuberculosis* Drives Rifampicin Resistance Codon Preference *in Vitro*

**DOI:** 10.1371/journal.pone.0029108

**Published:** 2012-01-03

**Authors:** Indra Bergval, Brian Kwok, Anja Schuitema, Kristin Kremer, Dick van Soolingen, Paul Klatser, Richard Anthony

**Affiliations:** 1 KIT Biomedical Research, Royal Tropical Institute, Amsterdam, The Netherlands; 2 Tuberculosis Reference Laboratory, Centre for Infectious Disease Control, National Institute for Public Health and the Environment (RIVM), Bilthoven, The Netherlands; 3 Departments of Microbiology and of Pulmonary Diseases, Radboud University Nijmegen Medical Centre/University Lung Centre Dekkerswald, Nijmegen, The Netherlands; St. Petersburg Pasteur Institute, Russian Federation

## Abstract

Both the probability of a mutation occurring and the ability of the mutant to persist will influence the distribution of mutants that arise in a population. We studied the interaction of these factors for the *in vitro* selection of rifampicin (RIF)-resistant mutants of *Mycobacterium tuberculosis*. We characterised two series of spontaneous RIF-resistant *in vitro* mutants from isoniazid (INH)-sensitive and -resistant laboratory strains and clinical isolates, representing various *M. tuberculosis* genotypes. The first series were selected from multiple parallel 1 ml cultures and the second from single 10 ml cultures. RIF-resistant mutants were screened by Multiplex Ligation-dependent Probe Amplification (MLPA) or by sequencing the *rpoB* gene. For all strains the mutation rate for RIF resistance was determined with a fluctuation assay. The most striking observation was a shift towards *rpoB*-S531L (TCG→TTG) mutations in a panel of laboratory-generated INH-resistant mutants selected from the 10-ml cultures (*p*<0.001). All tested strains showed similar mutation rates (1.33×10^−8^ to 2.49×10^−7^) except one of the laboratory-generated INH mutants with a mutation rate measured at 5.71×10^−7^, more than 10 times higher than that of the INH susceptible parental strain (5.46–7.44×10^−8^). No significant, systematic difference in the spectrum of *rpoB*-mutations between strains of different genotypes was observed. The dramatic shift towards *rpoB*-S531L in our INH-resistant laboratory mutants suggests that the relative fitness of resistant mutants can dramatically impact the distribution of (subsequent) mutations that accumulate in a *M. tuberculosis* population, at least *in vitro*. We conclude that, against specific genetic backgrounds, certain resistance mutations are particularly likely to spread. Molecular screening for these (combinations of) mutations in clinical isolates could rapidly identify these particular pathogenic strains. We therefore recommend that isolates are screened for the distribution of resistance mutations, especially in regions that are highly endemic for (multi)drug resistant tuberculosis.

## Introduction

The emergence, spread and persistence of drug resistance inhibits the successful treatment and control of tuberculosis (TB).

In contrast to many other bacterial pathogens, the etiological agent of tuberculosis, *Mycobacterium tuberculosis*, does not acquire antimicrobial resistance via horizontally transferred plasmids or other mobile genetic elements, but almost exclusively via the acquisition of point mutations or, occasionally, through genomic deletions [1], [2]. *M. tuberculosis* is thus a genetically isolated and clonal organism [1], [3] and recently acquired mutations are passed on to the progeny, resulting in the accumulation of mutations in the genome. Whether these mutations are sustained in the bacterial population depends on chance, their relative fitness and subsequent selective sweeps.

Genetic characterisation of drug-resistant clinical isolates indicates that only a small range of mutations is responsible for the majority of resistance in clinical isolates; screening for only 3 to 5 mutations in the *rpoB* gene detects more than 80% of all clinical *M. tuberculosis* isolates with resistance to rifampicin (RIF), a critical component of any successful anti-TB treatment and a marker for multidrug resistance [4], [5], [6], [7]. The majority of these mutations are located within an 81-bp region of *rpoB*, the gene that encodes the β-chain of RNA polymerase [8]. This mutational hotspot is therefore often referred to as the rifampicin resistance determining region. Virtually the same distribution of mutations conferring RIF resistance is seen in *in vitro* mutants [9], [10], [11], [12], [13]. RIF resistance is therefore often used as a proxy to study antibiotic resistance in the laboratory.

Not all *rpoB* mutations are equivalent; the implications for the bacteria, such as the level of RIF resistance or the consequential fitness, can vary considerably. Some mutations, like *rpoB*-S531L are found more often than others and detection of unusual or rare mutations in clinical isolates may therefore be indicative of distinctive circumstances.

In an earlier study, we found that acquisition of rifamycin resistance by a RIF-resistant, but rifabutin-susceptible strain, carrying an *rpoB*-S522L (TCG→TTG) mutation, was associated with a shift in the mutational spectrum outside of the rifampicin resistance determining region [9]. A possibly related effect was observed by others in a strain that caused an outbreak in London, which was resistant to isoniazid and became resistant to RIF via unusual mutations such as *rpoB*-V176F [14]. Also, a preference for specific drug resistance mutations in clinical isolates belonging to certain *M. tuberculosis* genotypes or strains obtained from specific geographical locations has been put forward [15], [16], [17], [18].

Together these data suggest that the genetic background of the *M. tuberculosis* bacteria, such as the genotype or pre-existing drug resistance, influence the optimal evolutionary route of the bacteria [19], [20], [21]; certain combinations of mutations may be lethal or lead to very unfit organisms, requiring adaptive mutations to restore their fitness. Experiments performed *in vitro* suggest that *rpoB* mutations S531L (TCG→TTG) and H526D (CAC→GAC) confer the lowest fitness deficit on the bacteria [8], [10], [11], [19], [22], [23]. However, methods by which “fitness” is measured in different studies, and even how fitness is defined, are not standardised [23], [24].

The predominance of certain *rpoB* mutations, in isolates of a specific bacterial genotype, is thus presumably due to a combination of the likelihood of each mutation occurring and its subsequent ability to survive and disseminate. Here we attempt to study the contribution and interaction of these two factors by determining the spectrum of spontaneous mutations in a well-characterised panel of laboratory and clinical strains using two different selection strategies. This panel includes strains representing genotypes from different geographical locations as well as several isoniazid-resistant isolates.

**Figure 1:**
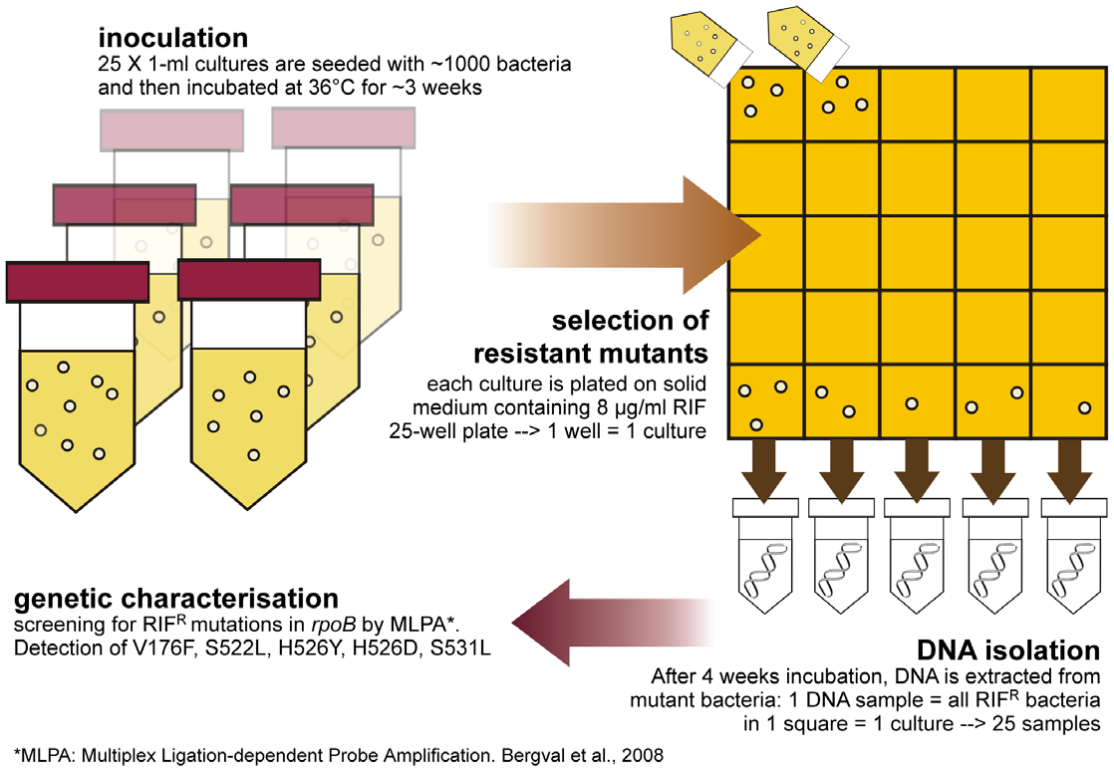
Graphic representation of the experimental procedures of method 1. For each strain 25 1-ml cultures were inoculated with approximately 1000 bacteria from a 10-ml starting culture after which they were incubated at 36°C in a shaking incubator. When the bacteria reached the mid-logarithmic phase (ca. three weeks), as determined by addition and colour development of the growth indicator resazurin, bacteria were transferred to rifampicin-containing solid medium to select for resistant mutants; the total contents of all 1-ml cultures were each plated on a single well of a 25-well plate, so that all 1-ml cultures from a single strain were plated on a single plate. The plates were then sealed and incubated at 36°C until sufficient bacterial growth of the mutants was visible (ca. four weeks). From each well, a DNA extract was made, so that one DNA sample contained DNA from any colonies that grew on the corresponding well. Finally, DNA samples were screened by MLPA [25] or sequencing of the rifampicin resistance determining region in rpoB.

**Figure 2:**
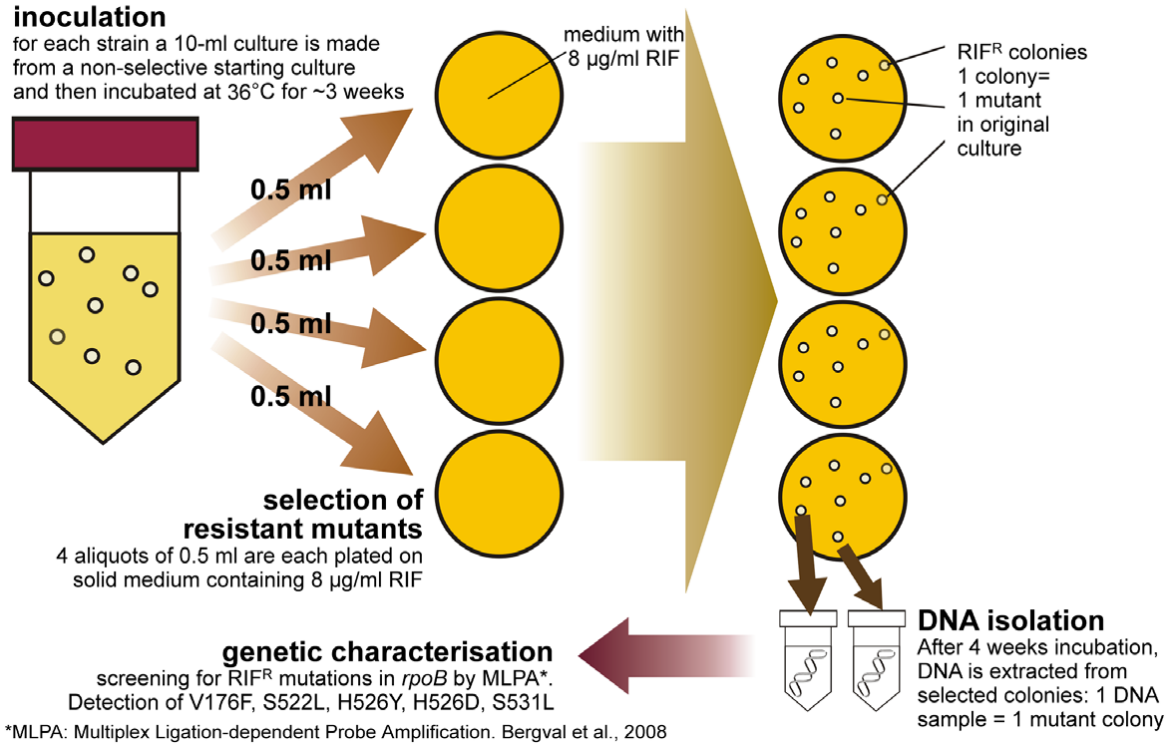
Graphic representation of the experimental procedures of method 2. For method 2 the same non-selective starting cultures as for method 1 were used to inoculate one 10-ml culture for each strain used in this study. These cultures were incubated at 36°C in a shaking incubator until mid-logarithmic phase (ca. three weeks). Then four aliquots of 0.5 ml from each culture were plated on rifampicin-containing solid medium to select for resistant mutants. The plates were sealed and incubated at 36°C until sufficient bacterial growth of the mutants was visible (ca. four weeks) When mutant colonies were visible on the plates, DNA was extracted: one mutant colony corresponded to one DNA sample. Finally, DNA samples were screened by MLPA [25] or sequencing of the rifampicin resistance determining region in rpoB.

Spontaneous rifampicin-resistant mutants from genetically characterised strains were selected from bacterial populations obtained from two different culture strategies; Method 1 (Figure 1) allowed us to observe the emergence of each targeted mutation with minimal competition between mutants, and with Method 2 (Figure 2) we determined the frequency, and thereby the “success”, of each mutation over time in a larger population. Mutants were characterized by Multiplex Ligation-dependent Probe Amplification (MLPA [25]), enabling detection of the RIF-resistance conferring mutations in *rpoB* V176F (GTC→TTC), S522L (TCG→TTG), H526Y (CAC→TAC), H526D (CAC→GAC) and S531L (TCG→TTG). Resistant mutants obtained without these mutations had two regions of their *rpoB* gene sequenced using methods previously published [9], [25].

Our results indicate that the (rapid) accumulation of drug resistance mutations can significantly reduce the subsequent spectrum of mutations. If so, these constrained pathways may also be applicable *in vivo*, which could facilitate the rapid detection of specific bacterial pathogens by molecular screening methods.

## Results

We set out to study the influence of the genotype of *M. tuberculosis* on the emergence and distribution of subsequent adaptive, spontaneous resistance mutations. Besides the influence of different genotypes, we also studied the influence of pre-existing isoniazid resistance, by comparing the spectrum of mutations acquired by isoniazid-resistant strains to the spectrum acquired by their (pan)susceptible parental strains. We used two different methods to map these *de novo* mutant distributions, which allowed us to study two distinct factors of acquisition and accumulation of mutations *in vitro*. In addition, we determined the mutation rate of all strains selected for this study.

### Recently acquired isoniazid resistance caused constrained genetic pathways *in vitro*

Six isoniazid (INH)-resistant strains, carrying different drug-resistance mutations, and their two parent strains were used for these experiments; laboratory-generated mutant strains H15, H26, H48, H71, H103 and their parent strain MTB72, and clinical isolate 2001–2184 and its isogenic INH-resistant clinical isolate 2001–2185 (Table 1). The number and nature of *rpoB*-mutants observed for each strain are depicted in Tables 2 (method 1) and 3 (method 2). No resistant mutants were obtained for strains H15, H26, H48 and H71 using method 1.

**Table 1:** Description of M. tuberculosis strains used in this study.

strain	DST (HR)	Genotype	origin
17583	SS	Beijing ‘atypical’ sublineage	RIVM [63]
2002-1640	SS	Beijing ‘atypical’ sublineage	RIVM
2002-1585	SS	Beijing ‘typical’ sublineage (*ogt*-GGG12GGA, *mutT2*-GGA58CGA, *mutT4*-CGG48GGG)	RIVM
9500592	SS	Beijing ‘typical’ sublineage (*ogt*-GGG12GGA, *mutT2*-GGA58CGA, *mutT4*-CGG48GGG)	RIVM [63]
2001–2184	SS	T1	RIVM (patient X)
2001–2185	RS	T1, *katG*-AGC315ACC	RIVM (patient X, 320 days later)
9900098	SS	LAM (Ag85C-GAG103GAA)	RIVM
2001-1669	RS	LAM (Ag85C-GAG103GAA), *katG*- AGC315ACC	RIVM (patient Y)
2001-1670	RS	LAM (Ag85C-GAG103GAA), *katG*- AGC315ACC	RIVM (patient Y, 225 days later)
MTB72	SS	Haarlem (*ogt*-ACC15AGC)	Laboratory strain [9]
H15	RS	Haarlem (*ogt*-ACC15AGC), *katG*-TGG321CGG	Derived from MTB72, selected with 20 µg/ml INH [2]
H26	RS	Haarlem (*ogt*-ACC15AGC), Δ*katG*(315+463)	Derived from MTB72, selected with 1 µg/ml INH (+H_2_O_2_) [2]
H48	RS	Haarlem (*ogt*-ACC15AGC), Δ*katG*(463)	Derived from MTB72, selected with 1 µg/ml INH [2]
H71	RS	Haarlem (*ogt*-ACC15AGC), Δ*katG*(315)	Derived from MTB72, selected with 20 µg/ml INH (+H_2_O_2_) [2]
H103	RS	Haarlem (*ogt*-ACC15AGC), *katG*-ACT271ATT	Derived from MTB72, selected with 0.4 µg/ml INH [2]

*Strains are identified by the name given by either the RIVM (numerical codes) or the KIT (letter+number). DST: drug susceptibility profile, H: isoniazid, R: rifampicin, S: susceptible, R: resistant. The genotype of the strains indicated in the table is determined by spoligotyping, mutations in parentheses are characteristic genotypic mutations identified by MLPA and confirmed by sequencing (ogt, mutT2, mutT4, Ag85C). Mutations in katG confer resistance to isoniazid; deletions were initially picked up by two different PCR reactions, amplifying either the region that covers the drug resistance mutations at codon 315 or the genotypic mutation at codon 463. The notation in this table indicates that the PCR fragment in question was absent and that therefore the region was deleted in the specific strain* [2].

**Table 2:** Spectrum of spontaneous rpoB-mutations obtained by method 1 (multiple parallel 1-ml cultures).

	RIF -resistance conferring mutation (codon change) in *rpoB*								
strain	V176Fn (%)	S522Ln (%)	H526Dn (%)	H526Yn (%)	S531Ln (%)	other *rpoB*n (%)	total	mutations in ‘other *rpoB*’ (n)	no mutation found (n)
MTB72	0 (0)	6 (16)	6 (16)	9 (24)	13 (34)	4 (11)	**38**	526CGC (3), 526CCC (1)	2
MTB72	0 (0)	1 (4)	11 (44)	3 (12)	5 (20)	5 (20)	**25**	513GAA (1), ins (dup 514–515), 526CGC (2), 526CCC (1)	2
MTB72	0 (0)	3 (13)	6 (26)	5 (22)	5 (22)	4 (17)	**23**	526CGC (3), 531TGG (1)	0
H103	0 (0)	1 (5)	2 (11)	6 (32)	6 (32)	4 (21)	**19**	526CCC (2), 526CGC(1), 531TGG (1)	2
2001–2184	0 (0)	2 (11)	4 (22)	4 (22)	4 (22)	4 (22)	**18**	526CGC (4)	3
2001–2185	0 (0)	0 (0)	2 (9)	8 (36)	5 (23)	7 (32)	**22**	522TGG (1), 526CGC (4), 526CCC (1), Δ526–527 (1)	4
9900098	0 (0)	6 (33)	2 (11)	1 (6)	2 (11)	7 (39)	**18**	513GAA (1), 526CGC (4), 527CAG (1), 526CCC (1)	7
2001-1669	0 (0)	3 (14)	2 (10)	6 (29)	1 (5)	9 (43)	**21**	526CGC (5), Δ524–527 (1), 533CCG (3)	8
2001-1670	1 (20)	1 (20)	0 (0)	1 (20)	1 (20)	1 (20)	**5**	533CCG (1)	20
9500592	0 (0)	4 (25)	2 (13)	8 (50)	0 (0)	2 (13)	**16**	526CGC (2)	0
2002-1640	3 (25)	0 (0)	2 (17)	2 (17)	4 (33)	1 (8)	**12**	526CGC (1)	0
2002-1585	0 (0)	3 (15)	1 (5)	7 (35)	5 (25)	4 (20)	**20**	513GAA (2), 516GTC (1), 531TGG (1)	0
17583	0 (0)	5 (21)	4 (17)	8 (33)	4 (17)	3 (13)	**24**	516 GTC (1), 519AAA (1), 526CGC (1)	0
**Total**	**4**	**35**	**44**	**68**	**55**	**55**	**261**		

*For strains H15, H26, H48 and H71 no mutants were obtained.* *Results for MTB72 (first row) and strains H103, 2001–2184 and 2001–2185 are obtained in the first experiment, where we assessed the influence of pre-existing INH resistance on the spectrum of mutations.* *Results for MTB72 (second row) and 9900098, 2001-1669 and 2001-1670 were obtained in the second experiment, where we determined the role of the LAM genotype on the spectrum of rpoB-mutations.* *Results for MTB72 (third row) and 9500592, 2002-1640, 2002-1585 and 17583 were obtained in the third experiment, where we determined the role of the Beijing genotype on the spectrum of rpoB-mutations.*

*ins: insertion, dup: duplication, Δ: deletion.*

**Table 3:** Spectrum of spontaneous rpoB-mutations obtained by method 2 (single colonies from 10-ml cultures).

	RIF -resistance conferring mutation (codon change) in *rpoB*								
strain	V176Fn (%)	S522Ln (%)	H526Dn (%)	H526Yn (%)	S531Ln (%)	other *rpoB*n (%)	total	mutations in ‘other *rpoB*’ (n)	no mutation found (n)
MTB72	0 (0)	2 (3)	10 (15)	20 (31)	27 (42)	6 (9)	**65**	526CGC (3), 526CCC (1), 531TGG (1), 513GAA (1),	1
MTB72	0 (0)	11 (39)	8 (29)	2 (7)	4 (14)	3 (11)	**28**	513GAA (2), 526CGC (1)	2
MTB72	0 (0)	2 (7)	5 (18)	4 (14)	4 (14)	13 (46)	**28**	513GAA (2), 526CGC (3), 526CCC (1), 529CTA (2), 531TGG (5)	0
H15	0 (0)	0 (0)	0 (0)	0 (0)	2 (100)	0 (0)	**2**	-	1
H26	0 (0)	0 (0)	1 (33)	0 (0)	2 (67)	0 (0)	**3**	-	1
H48	0 (0)	0 (0)	1 (20)	0 (0)	4 (80)	0 (0)	**5**	-	0
H71	0 (0)	0 (0)	0 (0)	0 (0)	19 (86)	3 (14)	**22**	Δ515–517 (1), 526CGC (1), 531CAG (1),	0
H103	0 (0)	0 (0)	2 (4)	7 (14)	34 (69)	6 (12)	**49**	513GAA (1), 522TGG (1), 526CCC (1), 526CGC (2), 531TGG (1)	0
2001–2184	0 (0)	0 (0)	0 (0)	3 (27)	4 (36)	4 (36)	**11**	522TGG (1), 526CCC (1), 526CGC (2)	1
2001–2185	0 (0)	0 (0)	3 (18)	7 (41)	7 (41)	0 (0)	**17**	-	1
9900098	3 (14)	1 (5)	6 (29)	7 (33)	1 (5)	3 (14)	**21**	513GAA (1), 526CGC (2)	1
2001-1669	0 (0)	5 (22)	3 (13)	8 (35)	2 (9)	5 (22)	**23**	513GAA (2), 519AAA (1), 529CTA (1), 533CCG (1)	0
2001-1670	6 (38)	0 (0)	2 (13)	2 (13)	4 (25)	2 (13)	**16**	526 CGC (2)	7
9500592	0 (0)	0 (0)	0 (0)	0 (0)	2 (29)	5 (71)	**7**	522TGG (2), 526CGC (3)	0
2002-1640	1 (3)	4 (13)	1 (3)	7 (23)	5 (16)	13 (42)	**31**	indel Δ512–519 ins ATC (1), ins 514–515 AAATTC (2), Δ517 (2), 513CTA (1), 526CCC (1), 526CGC (5), 531TGG (1)	0
2002-1585	0 (0)	2 (11)	5 (26)	1 (5)	4 (21)	7 (37)	**19**	513GAA (1), 522TGG (1), 526CGC (4), 531TGG (1)	0
17583	0 (0)	0 (0)	1 (20)	0 (0)	1 (20)	3 (60)	**5**	513GAA (1), 526CGC (1), 531TGG (1)	0
**Total**	**10**	**27**	**48**	**68**	**126**	**73**	**352**		

*Results for MTB72 (first row) and strains H103, 2001–2184 and 2001–2185 were obtained in the first experiment, where we assessed the influence of pre-existing INH resistance on the spectrum of mutations.* *Results for MTB72 (second row) and 9900098, 2001-1669 and 2001-1670 were obtained in the second experiment, where we determined the role of the LAM genotype on the spectrum of rpoB-mutations.* *Results for MTB72 (third row) and 9500592, 2002-1640, 2002-1585 and 17583 were obtained in the third experiment, where we determined the role of the Beijing genotype on the spectrum of rpoB-mutations. ins: insertion, indel: combined insertion/deletion, Δ: deletion.*

It was assumed that no *rpoB*-mutants were present at the time of inoculation and approximately 10^7^ bacteria were present at the time of plating, however, multiple mutational events occurred in some of the 1-ml cultures (indicated by the presence of more than one *rpoB* mutation identified by MLPA/sequencing). For pragmatic reasons, these were scored as two separate “counts”, therefore the number (n) in Table 2 does not represent the number of independent cultures where the specific mutation arose. In stead, it indicates the number of times the mutation was found and it should be noted that with our method it could not be determined whether all mutants in one culture carrying the same mutation were the result of one or more mutational events. We have shown previously that mixtures of genotypes can be detected with MLPA, to at least 1∶10 ratios [2]

The (Pearson's) Χ^2^ test was used to determine the probability of two hypotheses:

*h*_0a_: the rate of each of the four most prevalent mutations (S531L, H526D, H526Y and S522L) is equal

and

*h*_0b_: the distribution of *rpoB* mutations is unaffected by the presence of INH-resistance.

The results of this test are reported in Tables 4 and 5. Hypotheses were rejected at a *p*-value of 0.05 or lower.

**Table 4:** Probability of an equal distribution of the four targeted rpoB mutations in various M. tuberculosis strains.

strain	Probability	
	method 1 (1-ml)	method 2 (10-ml)
MTB72	0.30-0.20	**<0.001**
MTB72	**0.02-0.01**	0.06-0.05
MTB72	0.90-0.80	0.80-0.70
H15	NA	0.15-0.10
H26	NA	0.30-0.20
H48	NA	**0.04-0.03**
H71	NA	**<0.001**
H103	0.15-0.10	**<0.001**
2001–2184	0.90-0.80	0.07-0.06
2001–2185	**0.03-0.02**	**0.05-0.04**
9900098	0.15-0.10	**0.05-0.04**
2001-1669	0.20-0.15	0.30-0.20
2001-1670	0.90-0.80	0.20-0.15
9500592	**0.02-0.01**	0.15-0.10
2002-1640	0.30-0.20	0.30-0.20
2002-1585	0.20-0.15	0.40-0.30
17583	0.60-0.50	0.60-0.50

*The probability (p_a_) of hypothesis h0_a_ (the targeted rpoB-mutations (S531L, H526D, H526Y and S522L) have an equal chance of occurring) was determined by X^2^ for each strain for both method 1 and method 2. For all tested strains, except in the first experiment with MTB72, the statistical power of the X^2^ test was reduced due to one or more mutations occurring less than five times. NA: not available, mutants were not obtained for these strains.* ***Bold*** *: p_a_<0.05, therefore hypothesis is rejected.*

**Table 5:** Probability of the spectrum of rpoB mutations in the INH-resistant mutant and the wildtype parent being identical.

	Probability	
strain	method 1 (1-ml)	method 2 (10-ml)
H26	NA	0.60-0.50
H15	NA	0.5
H48	NA	0.40-0.30
H71	NA	**<0.001**
H103	0.60-0.50	**<0.001**
2001–2185	0.09-0.08	0.90-0.80
2001-1669	**<0.001** *	**0.01-0.001** *
2001-1670	0.40-0.30*	**<0.001** *

*The probability of hypothesis h0_b_ (the spectrum of rpoB-mutations of the INH-resistant mutant is not different than that of the wildtype parent) determined by X^2^ for all INH-resistant strains for both methods. For all of the tested strains the statistical power of the X^2^ test was reduced due to one or more mutations occurring less than five times. NA: not available, mutants were not obtained for these strains.*

*: since both 2001-1669 and 2001-1670 are resistant to INH (via katG-S315T), the spectrum was compared to the only susceptible LAM strain, 9900098. ***Bold*** *: p_a_<0.05, therefore hypothesis is rejected.*

With method 1 the proportions of the four targeted *rpoB*-mutations were not significantly different and *h*_0a_ was accepted for strains MTB72, H103 and 2001–2184 (*p*_a_-values 0.30-0.20, 0.15-0.10 and 0.90-0.80 respectively, Table 4). However, for strain 2001–2185 the *h*_0a_ null-hypothesis was rejected (*p*_a_<0.05, Table 4), indicating that the proportions of the four *rpoB*-mutants were not equal in this strain.

The results obtained by method 2 are depicted in Table 3. The S522L mutation was not observed in any of the strains, except for MTB72 (2/65 (3%)), while this mutation was found in 6/38 (16%, MTB72), 1/19 (5%, H103) and 2/18 (11%, 2001–2184) samples tested with method 1 (Table 2). Instead, most of the RIF-resistant mutants carried the *rpoB*-S531L mutation when method 2 was used; for all strains, except 2001–2185, which had acquired an equal number of H526Y mutants (7/17 (41%)), this was the category with the highest number of representatives (Table 3). For strains MTB72, H48, H71, H103 and 2001–2185 this shift in the mutational spectrum was significant; the *h*_0a_ was rejected for these strains, but not for H26, H15 and 2001–2184.

We also compared the mutant distributions obtained for the INH-resistant strains to the distributions obtained for the wildtype parent strains (Table 5). In comparison to their susceptible parent strains MTB72 and 2001–2184, strains H103 and 2001–2185 did not show a significantly different spectrum using method 1; *p_b_*-values were 0.60-0.50 for H103 and 0.09-0.08 for 2001–2185 (Table 5) and therefore *h*_0b_ was accepted. However, with method 2 the distribution of laboratory-generated mutants from H71 and H103 were dramatically different than that of parent strain MTB72; this difference was significant and *h*_0b_ was rejected (Table 3, *p*_b_<0.001 Table 5).

**Figure 3:**
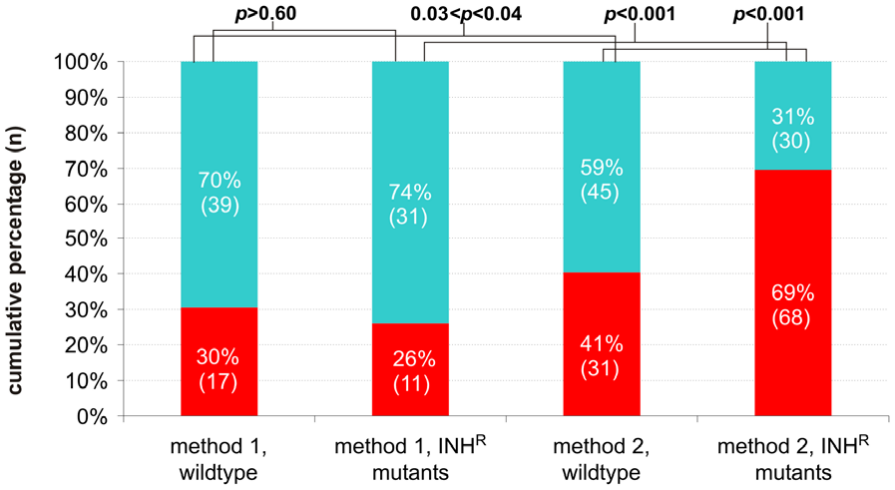
Proportion of rpoB-S531L and other mutations in rpoB acquired by INH-resistant M. tuberculosis strains compared to their susceptible parent strains. Results for all INH-resistant strains are grouped, as well as the results for are wildtype strains. Mutation distribution obtained for the INH-resistant group and the wildtype group and rpoB mutation distribution obtained for the two different experimental methods are compared. The red bars depict the proportion of rpoB-S531L mutations that were detected. The blue bars represent all other rpoB-mutations, whereas for the X^2^ test depicted in tables 4 and 5 only the four most prevalent mutations were taken into account. Numbers in the bar graphs represent the number of samples carrying the specific mutation as a percentage of the total amount of rpoB mutants and as absolute numbers (in brackets). The p-values above the bar graphs represent the probability that the distributions compared are similar.

Although the null hypotheses, *h*_0a_ and *h*_0b_, were accepted or rejected on the basis of the *p*-values, none of the strains, except MTB72 with method 1 (Table 2), met the criteria needed to ensure sufficient statistical power to the X^2^ test, possibly leading to a type II error (incorrectly accepting the null hypothesis). To investigate whether the shift in the spectrum of *rpoB*-mutations towards *rpoB*-S531L mutations was detectable with method 2 in both INH sensitive and all resistant strains, we decided to group the results obtained for all INH-susceptible strains (MTB72 and 2001–2184) and all INH-resistant strains (H15, H26, H48, H71, H103 and 2001–2185). We then compared the spectrum of mutations obtained by each of these two groups (mutants vs wildtype) between method 1 and method 2. Because we were only interested in the shift of the proportion of *rpoB*-S531L mutations, we reduced the groups of observed mutations to two: “*rpoB*-S531L” and “others”. The Χ^2^ test was then performed to test the similarity between these four mutant distributions obtained. Since there was only one degree of freedom (there are only 2 categories), Yates' correction factor was used (26). These results are depicted in Figure 3.

With method 1 30% (n = 17) of the mutants derived from the INH-susceptible parent strains had acquired an *rpoB*-S531L mutation. For the INH-resistant strains this was 26% (n = 11) of the total number of mutants. These proportions were almost the same and the difference between the wildtype and the INH-resistant strains was therefore not significant (*p*>0.60, Figure 3). In contrast, the spectrum of mutations obtained with method 2 resulted in 41% (n = 31) of *rpoB*-S531L mutants for the wildtype and 69% (n = 68) for the INH-resistant strains, which was a significant shift towards *rpoB*-S531L after acquisition of INH resistance (*p*<0.001, Figure 3).

We then compared the proportion of *rpoB*-S531L mutations acquired by INH-resistant strains via method 1 and method 2 (26% vs 69% respectively, Figure 3) and also found that the increased contribution of *rpoB*-S531L mutations was significant between the two methods (*p*<0.001, Figure 3). The mutation distribution of the wildtype pools had also significantly shifted towards *rpoB*-S531L (0.03<*p*<0.04), but to a lesser extent: 30% (n = 17) with method 1 and 41% (n = 31) with method 2, Figure 3).

These results suggest that pre-existing INH-resistance can considerably influence the spectrum of subsequent *rpoB* mutations, with a preference for *rpoB*-S531L mutations in the 10-ml cultures, but not in the 1-ml cultures.

Two of the Latin-American Mediterranean (LAM) strains, the paired isolates 2001-1669 and 2001-1670, carried an INH-resistance conferring mutation in the *katG* gene; S315T (AGC→ACC, Table 1). Resistance was, however, not acquired in the patient, since the mutation was already present at the moment of diagnosis (primary resistance). Both strains were already resistant to INH, therefore we could not determine the influence of the *katG*-S315T mutation on the spectrum of *rpoB*-mutations. In stead, we compared the spectrum of these two INH-resistant strains to the spectrum obtained by strain 9900098, the susceptible LAM strain in our test-panel. This strain showed a propensity for *rpoB*-S522L with method 1 (6/18 (33%), Table 2) and a preference for *rpoB*-H526D/Y with method 2 (13/21 (62%), Table 3). The spectrum obtained by strain 2001-1669 was significantly different from this spectrum, by both methods (*p*<0.001 in both cases, Table 5), but the spectrum obtained by 2001-1670 was only significantly different when method 2 was used (*p*<0.001, Table 3).

### The observed *in vitro* spectrum of mutations was not significantly correlated with the genotype of the strain

In addition to the influence of pre-existing INH-resistance we also assessed the spectrum of RIF-resistance conferring mutations of representative genotypes. We selected four strains with the Beijing genotype, two “atypical” Beijing strains (17583 and 2002-1640) and two “typical” Beijing strains (2002-1585 and 9500592) [27], [28], [29], and three LAM strains (9900098, 2001-1669 and 2001-1670), of which two were INH-resistant (Table 1). The same two methods, 1 and 2, for culture and selection of mutants were used as described earlier. Results are depicted in Tables 2 (method 1) and 3 (method 2). Although the *rpoB*-V176F was not included in the analyses with the Χ^2^ test, a separate column was included in the tables to indicate the proportion of this mutation that is only seldom identified in clinical isolates [23], [30]. The *rpoB*-V176F mutation is located outside of the 81-bp hotspot, where up to 96% of RIF-resistance conferring mutations appear in resistant strains, both *in vivo* as *in vitro* [4], [5], [8]. A higher prevalence of this mutation may indicate extraordinary circumstances [9].

Strain 2002-1640 had acquired the *rpoB*-V176F mutation at least three times independently with method 1, making it the second most frequent mutation in this strain (3/12 (25%), Table 2). Although the frequency of *rpoB*-V176F was lower, it was still observed in 2002-1640 with method 2 (1/31 (3%), Table 3). This rare mutation was also found in LAM-strain 2001-1670 (1/5 (20%), Table 2) with method 1 and with method 2 (6/16 (38%), Table 3) and in LAM-strain 9900098 (3/21 (14%), Table 3) with method 2 but not with method 1.

The *rpoB*-V176F was not observed in any of the RIF-resistant mutants derived from the other strains.

Another striking observation was that none of the 16 mutants derived from strain 9500592 obtained with method 1 had acquired an *rpoB*-S531L mutation, which is generally associated with the least fitness deficit and is most often seen in RIF-resistant clinical isolates. However, with method 2, 2/7 (29%, Table 3) mutants carried this mutation; the five other mutants carried uncommon mutations (Table 3). This shift away from *rpoB*-S531L observed with method 1 was significantly different from an equal distribution (*p*<0.02, Table 4) between the four most common *rpoB*-mutations, but the spectrum obtained with method 2 was not (0.10<*p*<0.15, Table 2).

As can be seen, there was a considerate amount of variation in the mutant distribution within as well as between the genotypes (Tables 2 and 3). Replication of the experiment with MTB72, a susceptible Haarlem strain, included in every experiment as a control, showed that the results between experiments were already quite different for a single strain (Tables 2 and 3), both with method 1 and method 2. Therefore, it was not possible to compare the mutant distributions between the different genotypes; combining the data per genotype would not have resulted in a representative distribution.

Furthermore, our data do not seem to show a clear systematic relationship between the amount (proportion), nature and spectrum of uncommon mutations in *rpoB* (“other *rpoB*”, Tables 2 and 3) and the genotype; any shift towards or inclusion of atypical *rpoB* mutations of a strain, appeared to be strain specific rather than genotype specific, as was the case for strains 2001-1669, 9900098 (method 1, Table 2), MTB72, H103, 95000592 and 2002-1640 (method 2, Table 3).

In our study no reproducible correlation between genotype and a specific mutation spectrum was observed.

### Clinical INH resistance did not lead to a significantly higher rifampicin mutation rate

**Figure 4:**
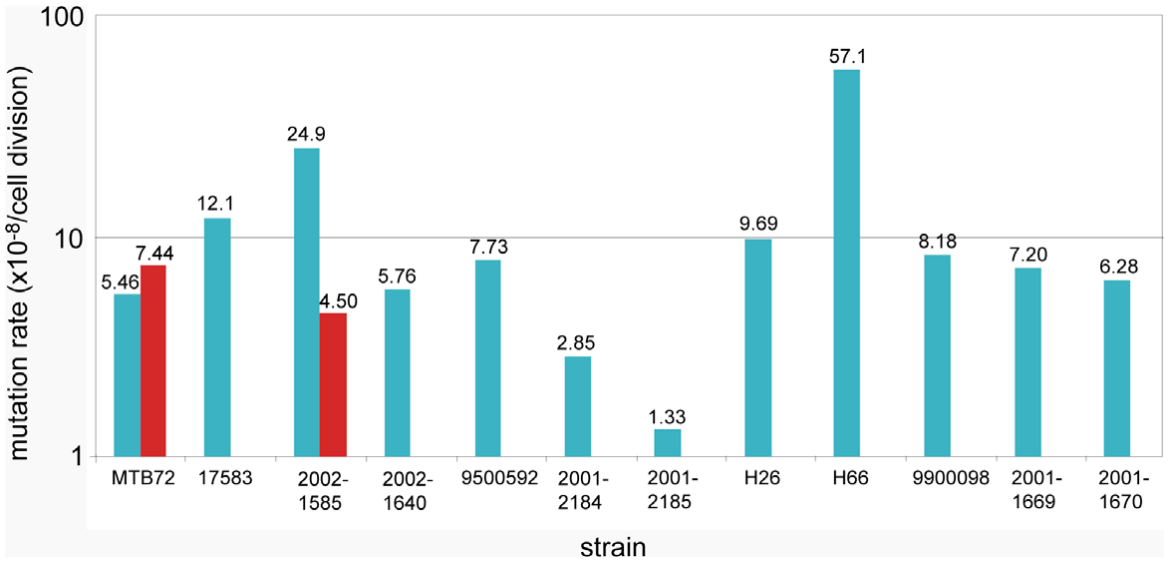
Mutation rates (×10^−8^/cell division), determined with 8 µg/ml rifampicin, for the M. tuberculosis strains used in this study. Description of the strains can be found in Table 1. Red bars indicate replicates from a second, independent experiment.

A fluctuation assay was performed to estimate the mutation rate to rifampicin resistance in the strains included in this study. Two independent experiments were performed for each strain, but, due to contamination and technical problems, duplicate results were only obtained for MTB72 and 2002-1585. Results are depicted in Figure 4.

Most of the mutation rates were measured at around 10^−8^, which is comparable to data we obtained previously [2] and to data published by others [13], [30]. Strains 17583 and 2002-1585 showed a moderately increased mutation rate (1.21×10^−7^ and 2.49×10^−7^, respectively; Figure 4), however, well within the range of natural or experimental variation; Werngren and Hoffner [13] have seen comparable variation when testing multiple strains and upon repetition of the experiment with strain 2002-1585 we observed a lower mutation rate (4.50×10^−8^, Figure 4). Although still within the limits, the isogenic strains 2001–2184 and 2001–2185, which represent the T1 spoligotype-family, have a somewhat lower mutation rate (2.85×10^−8^ and 1.33×10^−8^, Figure 4). However, strain H66, which obtained INH-resistance by a partial deletion of the *katG* gene, showed an increased mutation rate, when compared to its wildtype parent strain MTB72; under the same experimental conditions the increase was 10.5 times (5.71×10^−7^ versus 5.46×10^−8^, Figure 4). In contrast, for the strains which had acquired INH resistance in the patient, via *katG*-S315T (2001–2185, 2001-1669 and 2001-1670, Figure 4), we did not find an increased mutation rate.

## Discussion

The predominance of a mutation is the product of the probability of the mutation occurring in the genome and the probability of the altered microorganism surviving in a given environment. Thus, what ultimately matters for the generation of drug resistance is not the mutation rate, but the substitution rate; the rate at which a bacterial strain can produce viable, adequately adapted offspring. In this study we have shown that the *in vitro* mutation rate was within the same range for all but one *M. tuberculosis* strain tested and that there were no consistent significant differences in the spectrum of *rpoB*-mutations between the various *M. tuberculosis* genotypes we tested. However, the substitution rate may be different for the different strains; under more rigorous selective conditions, for example in the host, the type of resistance mutation in combination with the genetic background of a strain may have a more dramatic impact on which and how many mutants will survive and be transmitted to new hosts [21], [31], [32].

Previously, researchers have shown that drug resistance mutations can have varying consequences on the fitness of the bacteria carrying them [19], [20], [22], [23], [33], [34], [35]. This partly depends on the magnitude of the fitness deficit of each *de novo* resistance mutation, but can also be influenced by the genetic background of the bacterial strain and by the ability/opportunity to acquire compensatory mutations [19], [20], [36]. Most studies have shown that the S531L mutation in *rpoB* is the most prevalent rifampicin-resistance conferring mutation found *in vitro* and *in vivo*, presumably because it confers the least fitness deficit [8], [10], [11], [19], [22], [23], at least before transmission or adaptive mutation has occurred [37].

However, other studies have reported an altered distribution of mutations in *rpoB* among RIF-resistant isolates [9], [11], [15], [16], [17], [18], indicating that other resistance mechanisms can also be successful under certain circumstances.

In this study we hypothesized that the genetic background does not only influence the fitness of the bacteria, but also impacts the probability of specific mutations accumulating in a population. In addition, we hypothesized that an altered spectrum of mutations may also lead to a changed mutation rate due to a changed adaptation strategy [9].

We tested these hypotheses by selecting spontaneous RIF-resistant mutants using two distinct selection strategies which allowed us to investigate the likelihood of specific mutations in *rpoB* occurring (method 1) versus the likelihood of surviving in the population (the *in vitro* fitness, method 2). We made use of well-characterised *M. tuberculosis* strains with different genetic backgrounds. Mutations were identified by MLPA [25] or by sequencing of two regions in *rpoB* [9], [38]. To assess the effect of genetic background on the mutation rate we performed a fluctuation assay, using the same concentration of RIF (8 µg/ml) to select for resistant mutants [2].

Comparison of the *rpoB* mutant distribution obtained from the wildtype parent strains to the *rpoB* mutant distribution obtained from the INH-resistant daughter strains, showed that acquisition of INH-resistance resulted in a dramatic shift toward *rpoB*-S531L mutations (Figure 3). This effect was most evident when method 2 was used to select for RIF-resistant mutants. The mutant distribution of this group was significantly shifted compared to the wildtype INH-susceptible pool with method 2 and to the INH-resistant pool with method 1 (Figure 3). The laboratory-generated INH-resistant mutants (H15, H26, H48, H71, H103) contributed mostly to this shift; of 81 *rpoB*-mutants selected with method 2, 61 (75%) carried the *rpoB*-S531L mutation. In contrast, the clinical INH-mutants (2001–2185, 2001-1669 and 2001-1670) did not show this dramatic shift; they acquired an *rpoB*-S531L mutation in 7/17 (41%), 2/23 (9%) and 4/16 (25%) RIF-resistant mutants, respectively. As a reference, from the RIF-resistant mutants derived from the INH susceptible parent strains, MTB72 and 2001–2184, 27/65 (42%) and 4/11 (36%) mutants had acquired the *rpoB*-S531L mutation, respectively (Table 3).

This difference in *rpoB* mutant spectrum may find its origin in the different INH-resistance conferring mutations carried by the two populations; the laboratory-generated *rpoB*-mutants carry INH-resistance-conferring mutations that have rarely or even never been observed in clinical isolates [2], whereas the clinical strains in our study (2001–2185, 2001-1669 and 2001-1670) all carry the most prevalent mutation seen in INH-resistant patient isolates, *katG*-S315T [4], [5]. This mutation is thought to confer only a very small fitness deficit [20], [34], [37], [39], [40] and the strains carrying this mutation therefore would have a higher “baseline fitness” than the strains carrying uncommon INH-resistance mutations. It is assumed that strains with mutations that confer little fitness costs are not in need of restoration of fitness and therefore not likely to acquire adaptive mutations [41]. As a consequence of the higher “baseline fitness”, *katG*-S315T mutants will be more able to withstand or overcome the deleterious effects associated with certain *rpoB*-mutations, allowing for a wider spectrum of RIF-resistance conferring mutations. Strains that already have a low “baseline fitness”, such as potentially our *in vitro* INH-mutants, can probably only survive acquisition of “fit” *rpoB*-mutations, such as *rpoB*-S531L. Thus, in our model the relative fitness cost of the different *rpoB* mutations is probably more apparent in strains that are already quite unfit.

The higher baseline fitness of the clinical INH-resistant mutants could, in part, also be a result of adaptive or compensatory mutations, acquired within the patient. A higher baseline fitness could have also allowed for the many rare mutations in *rpoB*, including deletions and insertions, we observed for almost all strains with both methods (Tables 2 and 3); most of these mutations are rarely or never found in RIF-resistant strains isolated from (immunocompetent) patients, where severe (sequential) bottlenecks have probably selected against most of these mutations.

Since multiple mutants are present with method 2 there is more competition between mutants than with method 1 [11], we therefore would have expected a reduced spectrum of *rpoB*-mutations. In addition, other researchers have reported *rpoB*-S531L to be the most frequently observed mutation in similar experiments [12]. In those experiments 5-ml cultures were used and there was no opportunity to identify multiple mutations in a whole culture, as we did; in stead one or sometimes 10 colonies per plate were analysed, perhaps biasing towards “fitter” and therefore bigger colonies to be picked.

Our results indicate that probably more severe bottlenecks or strong selective sweeps have to be applied *in vitro* to mimic the selection processes bacteria are constantly subjected to when living inside the host [36]. However, prolonged incubation of bacteria can already lead to a decreased spectrum of mutations [11], suggesting that despite an initial burst of multiple *rpoB*-mutations, even *in vitro* conditions can induce rather constrained evolutionary pathways [21].

We have shown that, regardless of genotype, many more drug resistance mutations occur than would be expected on the basis of clinical screening. Particularly codon 526 can be highly variable, as was observed by others [4], [5], [12], [22], and we detected many uncommon mutations (Tables 2 and 3). We also found a relatively high degree of insertions and deletions, which did not seem to be specific for any strain or method and there were some inclinations towards certain mutations, in particular for the rare mutation V176F. However, we did not detect a difference in the spectrum of mutations between the various genotypes. We did not detect a specific *M. tuberculosis* genotype-related *in vitro* effect that determines which mutations predominate.

Others have performed similar studies, albeit on a smaller scale and with more focus on Beijing/non-Beijing [10], [13], [24], and also did not find a genotype-related effect. We observed that the mutational spectrum can vary substantially between experiments. Researchers have previously reported that the spectrum of RIF-resistance conferring mutations in *rpoB* can be dependent on the growth conditions of the bacteria, such as the pH of the growth medium or even the age of a bacterial culture [11]. Genotype-related characteristics have been observed *in vivo* [15], [16], [17], [18] but to our knowledge structural differences between *M. tuberculosis* genotypes have yet to be demonstrated *in vitro*.

We have focused here on differences in evolutionary pathways of the bacteria, but are aware that differences observed *in vivo* to a certain extent may be attributable to host characteristics; reports of “favoured mutations” have often been restricted to a single geographical location, where it is likely that hosts share genotypic characteristics [42], [43]. It has furthermore been discovered that host factors, such as the Toll-like receptor 2 allele, can partly influence the manifestation of disease and the susceptibility of humans to certain bacterial genotypes [44].

In a recent study sequential, increasingly drug-resistant isolates were obtained from a single patient and it was shown that the first (susceptible) and the last (multidrug resistant) isolate only differed by the two drug resistance-conferring mutations, as determined by whole genome sequencing [32]. This homogeneity for INH and RIF resistance conferring mutations in sequential isolates from a single patient was corroborated by others [45]. These reports imply that the selection pressure exerted by antibiotic usage can significantly reduce the spectrum of viable mutants and evolutionary pathways are probably restricted as a result [21], [46]. However, host-to-host transmission probably has an even more severe selective and thus constraining effect [47], [48]. In addition, Schürch et al [49] have retrospectively followed a chain of transmission of TB in the Netherlands over a period of more than a decade; the strain in question only acquired six mutations, of which five had been acquired within one patient.

In areas where transmission is rapid, successful strains [50], [51] may have very different characteristics to strains which are successful in more slowly evolving epidemics [48]. These two forms of transmission could lead to very different evolutionary routes; in areas with a high transmission rate it is likely that clusters of drug-resistant strains will be clonal and acquisition of drug resistance happened only once (before the spread – secondary resistance). On the contrary, in regions where the epidemic moves more slowly, drug-resistant strains are, at least initially, not expected to be highly clustered since drug resistance is more likely to be established within the patient (primary resistance) and adaptive evolution appears to continue even during latent infection [52].

If bacteria with specific mutations have a significant advantage in a disseminating population, the same drug resistance mutations will have a higher chance of occurring repeatedly, such as *rpoB*-S531L in our *katG* mutants. Thus, such a cluster of primary resistant strains would be indistinguishable from a truly clonal cluster of secondary resistant strains by current genotyping methods.

This situation is analogous to the high prevalence of *rpoB*-S531L mutants in our 1-ml experiments (method 1) which are not clonal, whereas *rpoB*-S531L mutants from the 10-ml experiment (method 2) may (or may not) represent clonal expansion of a singe mutational event. Misinterpretation of these two scenarios may result in incorrect measures being taken and failure to control the spread of resistance.

It has been suggested that sequencing of complete bacterial genomes should be used for the standard screening of clinical isolates [53]. Whole genome sequencing of seemingly clonal drug resistance clusters could reveal additional (“piggy-back”) mutations, indicating if drug resistance was indeed spread or acquired within the host.

A recent article [6] reports that the sensitivity of a molecular drug resistance test depended on the resistance profile of the bacteria tested, varying from close to 91% for MDR-TB to only 56%/70% for strains with monoresistance to INH or RIF. These results are supported by Van Rie *et al.* who analysed multiple strains from South Africa and concluded that by targeting just three mutations it was possible to detect up to 90% of MDR-TB [7].

As most molecular tests target only the most prevalent drug resistance conferring mutations, the increased sensitivity for MDR strains may be a direct result of a constrained pathway for the bacteria: only a few combinations of drug resistance conferring mutations can lead to a “fit” MDR strain, whereas a monoresistant strain can have a much wider spectrum of mutations. Indeed, in a study performed by Iwamoto it was shown that as strains accumulate multiple drug resistance mutations, the spectrum of mutations is considerably reduced [54].

These reports, in addition to our data, indicate that sign epistasis can play a significant role in the development of multidrug resistance in *M. tuberculosis*, as was suggested by Trindade et al. [55] and that the routes to a successful MDR or even XDR strain tend to become more restricted with each mutation that is acquired [21].

We are entering an era in which we will recognise the importance of certain mutations and we can use them to build early warning systems or optimise treatment [31]. Although for a genetically stable organism such as *M. tuberculosis* only a fraction of the data generated will be informative, data acquired with whole genome sequencing will be invaluable for the design of molecular tests with a much higher discriminatory ability than current methods, by targeting informative single nucleotide polymorphisms scattered throughout the genome [2], [25], [53], [56], [57], [58], [59].

## Materials and Methods

### *M. tuberculosis* strains used

Fifteen strains were used during the course of this study, including clinical isolates with and without isoniazid (INH) resistance, and a laboratory strain (MTB72) and its spontaneous INH-resistant mutants. This collection of strains represents *the M. tuberculosis* genotypes Haarlem [60], Beijing (‘typical’ and ‘atypical’ sublineage [28], [29]), Latin-American Mediterranean (LAM) [61] and T1 [62]. *M. tuberculosis* strains 2001–2184, 2001–2185, 2002-1585, 2002-1640, 9900098, 2001-1669, 2001-1670, 17583 and 9500592 were acquired as pure cultures on slope from the RIVM in Bilthoven, the Netherlands. The *M. tuberculosis* strains 17583 and 9500592 were presented previously as reference Beijing strains by Kremer et al. [63]. Strains 2001–2184 and 2001–2185 are sequential isolates from the same patient (see Table 1); for this reason they are assumed to have the same clonal origin. The same is true for strains 2001-1669 and 2001-1670.

Strain MTB72 (ATCC 35801) is a pansusceptible laboratory strain belonging to the Haarlem genotype. Strains with a HXX code are spontaneous INH-resistant mutants, derived from strain MTB72 in our laboratory as described previously [2].

The characteristics of the strains and their origins are described in Table 1.

### Bacterial culture

Bacteria were cultured in Middlebrook 7H9 medium (Difco, BD, Sparks, MD, USA), supplemented with oleic acid/albumin/dextrose/catalase (OADC Enrichment, BD, Sparks, MD, USA), in a shaking incubator at 37°C. For all strains, a liquid starting culture was made by inoculating pure colonies from Löwenstein–Jensen or Coletsos slopes into 10 mL of liquid culture medium. When these cultures reached the logarithmic growth phase (circa 3 weeks) they were mixed vigorously to homogenize the bacterial suspension. Clumped cells were allowed to settle for 3 min and aliquots of the cell suspension were then transferred to fresh nonselective liquid medium to make multiple parallel cultures of 1 mL (method 1) or a final culture of 10 mL (method 2) as described below.

#### Method 1 (Figure 1)

For each strain 25 1-ml cultures were inoculated from the 10-ml non-selective liquid starting culture by transferring 1 uL of this cell suspension (∼1000 bacteria) to a 2 mL screwcap tube containing 1 mL of MB7H9 medium + OADC with a sterile inoculation needle. Two to three sterile glass beads were added to each culture to ensure mixing. The 1 mL cultures were incubated in a shaking incubator at 36°C for approximately three weeks. Bacterial growth was monitored each week by adding 30 µL of a 0.02% (wt/vol) resazurin solution (Sigma), a growth indicator, to additional 1-ml cultures incubated simultaneously. These additional cultures were used only to monitor growth and resazurin was not added to the cultures from which the RIF-resistant mutants were analysed. After addition of the resazurin, all cultures were wrapped in aluminum foil and after 24 hours incubation at 36°C the color development was determined. After sufficient growth, when the reference cultures turned a bright pink, the target cultures were centrifuged at 5000 *g* for 8 min and 850 µL of the supernatant was discarded. For each culture the remaining 150 µL was resuspended and plated in one well of a square 25-well replica plate (Greiner, Germany) containing 3 mL of MB7H11 + OADC supplemented with 8 mg/L rifampicin (Sigma–Aldrich Chemie) to select for RIF-resistant mutants.

The 25-well square plates were allowed to dry in a biosafety laminar flow cabinet, until all liquid was absorbed into the solid medium. The plates were then sealed in plastic bags and incubated at 36°C. After growth was clearly visible in all or most wells, total populations of mutants were isolated from each well and analysed for mutations in *rpoB* (see below).

#### Method 2 (Figure 2)

For each strain a single 10-ml culture was made with a 0.5 ml inoculum from the 10-ml, non-selective liquid starting culture described above. Bacterial growth was monitored by determining the turbidity of the cultures at regular intervals. When sufficient growth was established, from each strain four 0.5 ml aliquots were taken from each strain and plated on four separate 8 mg/L RIF-containing plates to select for resistant mutants. Plates were wrapped and sealed separately in plastic bags to minimise cross-contamination and incubated at 36°C for 3–4 weeks, until clear mutant colonies could be observed. DNA was then isolated from single colonies by the method described below and samples were further analysed to screen for mutations in *rpoB*.

### Screening/characterisation of mutants

#### Isolation of DNA

For method 1 200 µL lysis buffer (10 mM Tris-HCl/1 mM EDTA pH 8.0 containing 1% Triton X-100 (BDH Laboratory Supplies, Poole, England)) was placed in each well of the 25-well square plates. Resuspension of the mutant colonies was ensured by pipetting the buffer up and down with a micropipette. With a clean pipette filtertip the suspension containing the total mutant population from a single well, was transferred to a microcentrifuge tube and then heated at 95°C in a heat block for 30 minutes. After lysis, cells were centrifuged at 5000 *g* for 3 minutes and 130 µL of the supernatant was collected.

For method 2 individual mutant colonies were picked from the RIF-containing plate and each suspended in 150 µL lysis buffer after which the same procedure as described above for method 1 was followed to extract the DNA.

#### Multiplex Ligation-dependent Probe Amplification

Crude DNA samples were analysed by Multiplex Ligation-dependent Probe Amplification (MLPA), enabling detection of the RIF-resistance conferring mutations in *rpoB* V176F, (GTC→TTC), S522L (TCG→TTG), H526D (CAC→GAC), H526Y (CAC→TAC) and S531L (TCG→TTG). *M. tuberculosis-specific* MLPA was performed as previously published [25], except in the present study only mutations in *rpoB* are reported.

#### Sequence analysis rpoB

For all RIF-resistant mutants for whom no mutation in *rpoB* was identified by MLPA, two fragments of the *rpoB* gene were sequenced. The same DNA extracts used for MLPA were used for PCR and subsequent sequencing analysis. PCR of *rpoB* clusters I and III was carried out as previously described [9], using primer pairs rpoB-2F/2R and rpoB-7F/7R respectively [25]. The PCR products were sequenced in both directions using the dideoxy chain termination method with the Big Dye Terminator cycle sequencing Kit (Applied Biosystems). Sequence analysis was performed on a 310 Genetic Analyzer (Applied Biosystems). All *rpoB* codon numbers, except V176, are reported using the *E. coli* numbering system.

### Fluctuation assay

The mutation rates (i.e. the chance of a mutation occurring per generation) for rifampicin resistance were estimated with a fluctuation assay. Our method has been previously published [2] and was based on the *p*0-method described by Luria and Delbrück [64].

### Statistical analysis

For all analyses our null hypothesis (*h*_0_a) was that under each condition tested each mutation in *rpoB* was equally likely to occur and secondly (*h*_0_b) that the distribution of specific mutations would be unaffected by the presence of INH resistance. To test this hypothesis Pearson's Χ^2^ test was used. Hypotheses were rejected at a *p*-value of 0.05 or lower. We used the Χ^2^ test to determine if the frequency of each of the four most prevalent mutations (S531L, H526D, H526Y and S522L) was equal when using each selection method and secondly, to determine whether the distribution of *rpoB* mutations in each of the INH-resistant mutants was equal to that of the INH susceptible parent. The *p*-values of the distributions of the individual strains are depicted in Tables 4 and 5. In most of the cases the statistical power of the Χ^2^ test was reduced, since not all of the criteria for the Χ^2^-test were met (i.e. in all the observed categories the minimum sample size should be five and none of the observed frequencies should be zero). Therefore we grouped all strains which were INH-resistant and all strains which were INH-susceptible and reduced the categories to “*rpoB*-S531L” and “other mutations”. To adjust for this reduction in number of categories, we used Yates' correction for continuity [26].

### GenBank accession numbers

The majority of the rifampicin resistance-conferring mutations in *rpoB* we found in this study have been previously observed and reported. Those mutations that were not deposited to GenBank (http://www.ncbi.nlm.nih.gov/Genbank/) or uploaded to the *M. tuberculosis* drug resistance mutations database TBDReaMDB (http://www.tbdreamdb.com/) were regarded as novel and were deposited to GenBank. Only mutations found in DNA samples derived from single mutant colonies (method 2) and not from mixed cultures (method 1) were considered. Mutations in *rpoB* AAC519AAA (GenBank JN819066), CGA529CTA (GenBank JN819067), indel Δ512–519 ins ATC (GenBank JN819068) and ins 514–515 AAATTC (GenBank JN819069) were deposited to the GenBank database. In addition, the isoniazid resistance-conferring mutation *katG* ACT271ATT in strain H103 was deposited to GenBank, under the accession number JN819065.
